# Role of ventral medullary catecholaminergic neurons for respiratory modulation of sympathetic outflow in rats

**DOI:** 10.1038/s41598-017-17113-7

**Published:** 2017-12-04

**Authors:** Davi J. A. Moraes, Leni G. H. Bonagamba, Melina P. da Silva, Julian F. R. Paton, Benedito H. Machado

**Affiliations:** 1 School of Medicine of Ribeirão Preto, Department of Physiology, University of São Paulo, Ribeirão Preto, SP Brazil; 2 School of Physiology, Pharmacology and Neuroscience, Biomedical Sciences, University of Bristol, Bristol, UK; 3 Department of Physiology, Faculty of Medical and Health Sciences, University of Auckland, Auckland, New Zealand

## Abstract

Sympathetic activity displays rhythmic oscillations generated by brainstem inspiratory and expiratory neurons. Amplification of these rhythmic respiratory-related oscillations is observed in rats under enhanced central respiratory drive or during development of neurogenic hypertension. Herein, we evaluated the involvement of ventral medullary sympatho-excitatory catecholaminergic C1 neurons, using inhibitory Drosophila allatostatin receptors, for the enhanced expiratory-related oscillations in sympathetic activity in rats submitted to chronic intermittent hypoxia (CIH) and following activation of both peripheral (hypoxia) and central chemoreceptors (hypercapnia). Pharmacogenetic inhibition of C1 neurons bilaterally resulted in reductions of their firing frequency and amplitude of inspiratory-related sympathetic activity in rats in normocapnia, hypercapnia or after CIH. In contrast, hypercapnia or hypoxia-induced enhanced expiratory-related sympathetic oscillations were unaffected by C1 neuronal inhibition. Inhibition of C1 neurons also resulted in a significant fall in arterial pressure and heart rate that was similar in magnitude between normotensive and CIH hypertensive rats, but basal arterial pressure in CIH rats remained higher compared to controls. C1 neurons play a key role in regulating inspiratory modulation of sympathetic activity and arterial pressure in both normotensive and CIH hypertensive rats, but they are not involved in the enhanced late-expiratory-related sympathetic activity triggered by activation of peripheral or central chemoreceptors.

## Introduction

It has long been recognized that vasoconstrictor class of sympathetic fibers show respiratory-related oscillations^1–3^. This respiratory–sympathetic interaction underpins the production of rhythmic fluctuations in arterial pressure (Traube–Hering waves), which are derived from phasic constriction of the arterial tree^4–6^. An enhancement of these rhythmic oscillations is important for mediating sympatho-excitatory responses to cardio-respiratory reflex activation under increased central respiratory drive^2,7–9^ and crucial for the sympathetic over activity observed in animal models of hypertension^6,10,11^. Despite the clear importance of this rhythmic component to control the level of sympathetic activity and cardiovascular function, its central origin remains elusive.

Vasoconstrictor sympathetic preganglionic neurons located in the intermediolateral cell column (IML) of the spinal cord receive synaptic excitation from supraspinal pre-sympathetic neurons located in the rostral ventrolateral medulla (termed here RVLM neurons)^12–14^. The RVLM neurons comprise glutamatergic C1 (catecholaminergic) and non-C1 (non-catecholaminergic) phenotypes^15,16^ that are believed to contribute to the generation of sympathetic outflow, since bilateral inactivation of the RVLM reduces sympathetic activity and arterial pressure to levels similar to those observed after transection of the spinal cord, at least in the anaesthetized state^17,18^. RVLM C1 and non-C1 neurons are both modulated by respiration^19^ and both show increased respiratory-related activity during hypoxia or hypercapnia^20,21^. In this regard, our previous studies have demonstrated that rats submitted to chronic intermittent hypoxia (CIH) exhibit active expiration^10^ and an increase in the firing frequency of expiratory-modulated pre-sympathetic RVLM C1 neurons^19,22^, which was also reflected in sympathetic post-ganglionic activity^10^. Indeed, we proposed that this expiratory-related sympathetic activity may contribute to the sustained hypertension induced by CIH in rats^23^. Although depletion or acute silencing of the majority of C1 neurons reduced the sympatho-excitation induced by activation of peripheral^24^ or central^25^ chemoreceptors in rats, the contribution of C1 and non-C1 RVLM neurons for generating expiratory-related increases in sympathetic activity evoked by CIH or activation of peripheral or central chemoreceptors has not yet been characterized.

Herein, we acutely silenced C1 neurons by application of the insect peptide allatostatin (Alst) following cell-specific targeting with a lentiviral vector to express the inhibitory Drosophila Alst receptor (AlstR)^26^ in the RVLM of rats. Using both *in vivo* (conscious) and *in situ* (arterially perfused brainstem preparations) rats, we determined the contribution of the C1 neuronal population for the generation of expiratory-modulated sympathetic activity of rats under enhanced central respiratory drive induced by either CIH or activation of peripheral or central chemoreceptors.

## Results

### Location and efficacy of viral gene transfer

**Figure 1:**
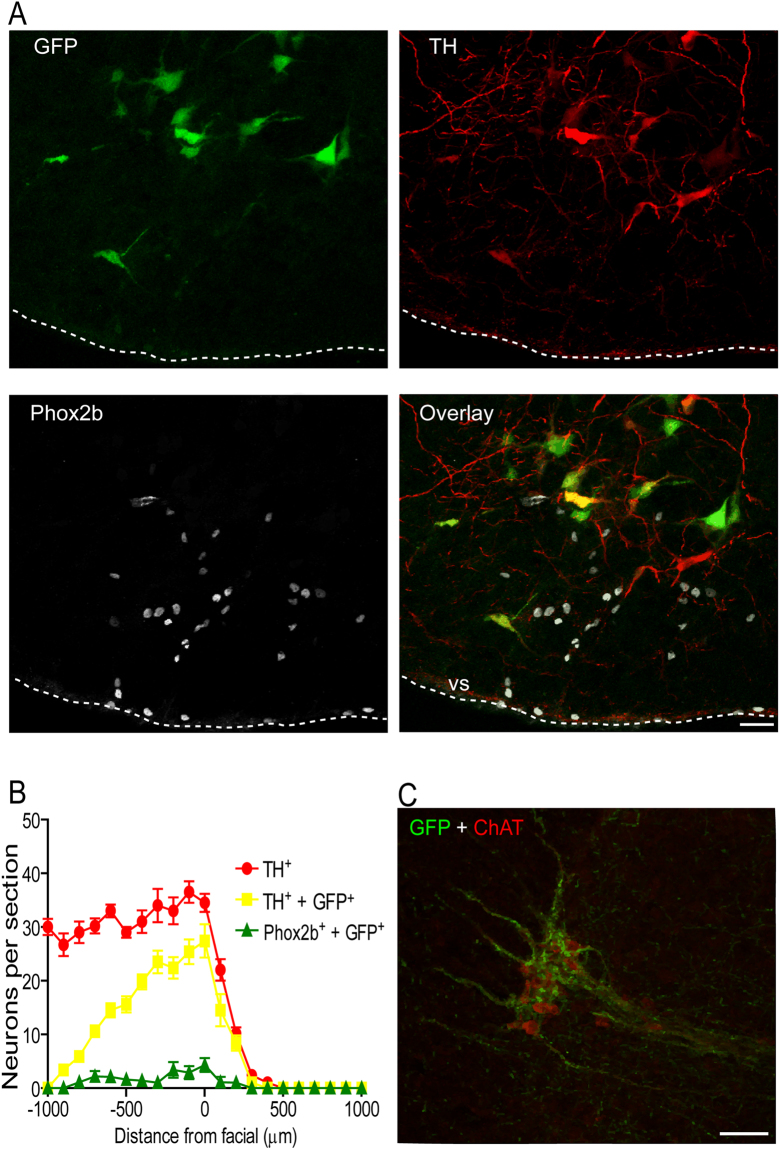
AlstR expression in C1 neurons. (**A**) Immunofluorescence images of a 40 μm coronal section showing AlstR-GFP immunoreactivity (green) in TH-immunoreactive (red) neurons in C1 group 15 days after PRSx8-AlstR-GFP-LVV injections. Note the high, but not absolute, specificity of the PRSx8 promoter in the merged images. Scale bar: 20 μm. Abbreviation: vs, ventral surface. (**B**) Neuron counts of total TH-immunoreactive/Phox2b-negative cells (TH^+^; red; C1 neurons), TH-immunoreactive/Phox2b-negative and AlstR-GFP-immunoreactive neurons (TH^+^  + GFP^+^, yellow), and Phox2b-positive AlstR-GFP-immunoreactive neurons (Phox2b^+^  + GFP^+^, green; non-C1 neurons) in the RVLM region after LVV injections. Numbers on the X axis indicate distances from caudal pole of facial nucleus [0]. (**C**) Spinal cord coronal section between T8–10 showing GFP-positive varicose axons (green) from C1 neurons throughout the IML closely opposed to sympathetic preganglionic ChAT-positive neurons (red). Scale bar: 50 μm.

Catecholaminergic brainstem neurons express transcriptional factor Phox2 and can be targeted using lentiviral vectors (LVV) to express a gene of interest under the control of an artificial promoter - PRSx8^25,27,28^. We examined the efficacy of viral-transduction of ventrolateral medullary C1 neurons by immunofluorescent co-localization of AlstR-GFP and tyrosine hydroxylase (TH) and absence of Phox2b expression (a marker of the ventral medullary CO_2_-sensitive neurons) after bilateral microinjections of PRSx8-AlstR-GFP-LVV (Fig. 1A). GFP immunostaining revealed bilateral clusters of AlstR-GFP-expressing RVLM neurons between 11.6 and 12.8 mm caudal to Bregma [200 µm rostral to 1000 µm caudal to the caudal pole of the facial nucleus (0); Fig. 1B]. Between 0–500 µm caudal to the facial nucleus, where most C1 pre-sympathetic neurons reside^29^, AlstR-GFP expression was found bilaterally in 69 ± 2% (n = 67 rats) of all identified C1 (TH-positive/Phox2b-negative) RVLM neurons, confirming effective targeting of the C1 neuronal population. We also found that C1 neurons projected to sympathetic preganglionic neurons in the thoracic (T8-T10) IML, as reflected by the distribution of varicosed axons intermingled with choline acetyltransferase (ChAT)-immunoreactive neurons (n = 4; Fig. 1C). Additional Phox2b-positive/GFP-expressing non-C1 neurons were scattered within ventral medulla beneath and caudal to the caudal border of the facial nucleus (5 ± 1% of GFP-expressing neurons) corresponding to the Retrotrapezoid Nucleus (RTN; Fig. 1B), as described before^30^. Only 1.9 ± 0.7% of GFP-expressing neurons in the ventral medulla lacked either TH or Phox2b expression (non-C1 neurons).

**Figure 2:**
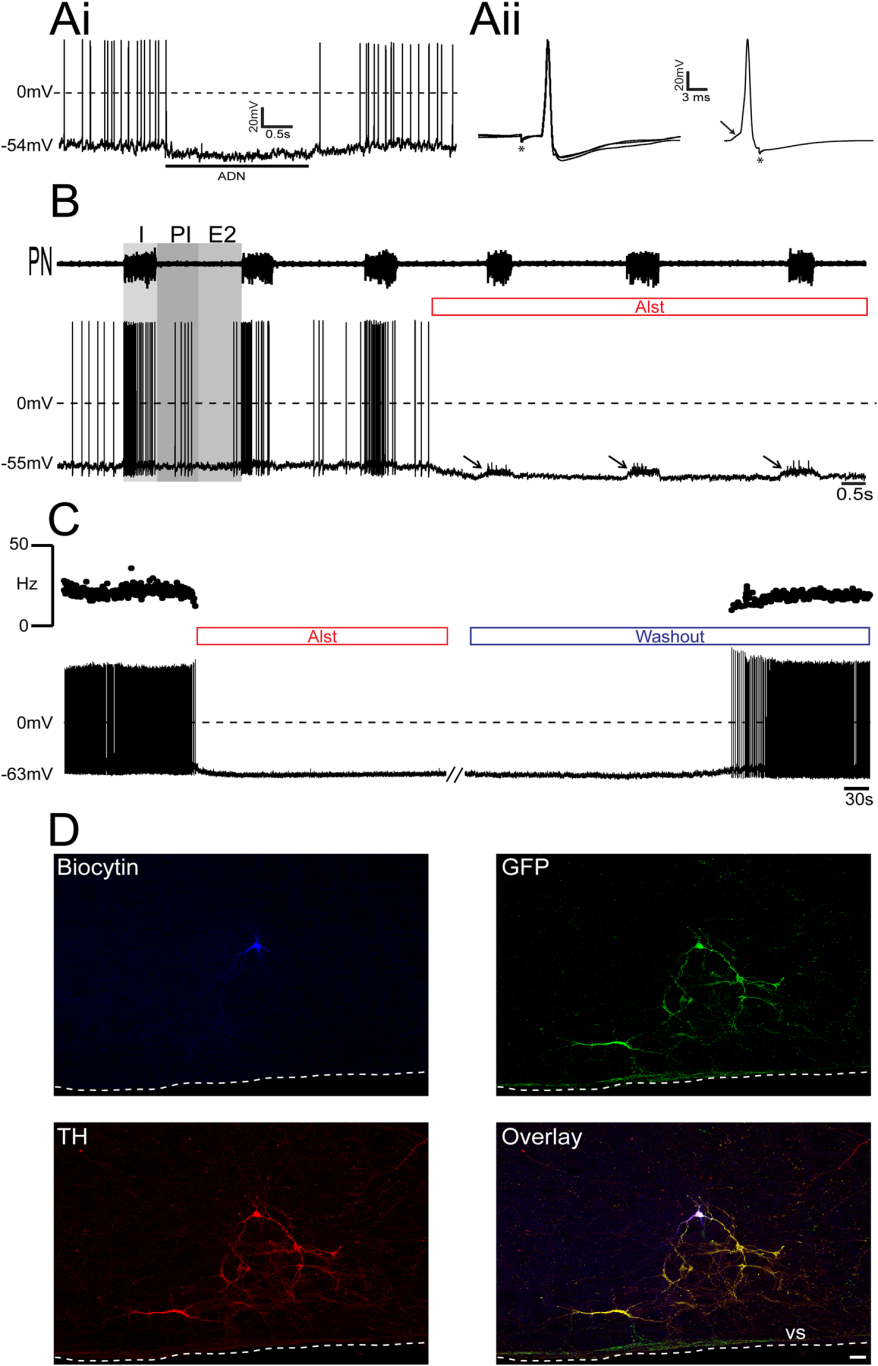
Effect of AlstR activation on the firing frequency of inspiratory-modulated RVLM pre-sympathetic neuron recorded *in situ*. (**Ai**) Representative RVLM pre-sympathetic neuron that was hyperpolarized and silenced by baroreflex activation [ipsilateral aortic depressor nerve (ADN) stimulation; 0.2 ms, 50 Hz, 2 s - indicated by bar]. (**Aii**) The same barosensitive pre-sympathetic neuron was antidromically activated from the IML (T8-T10) with constant latency (left). The antidromic spike was absent (top right) as the result of a collision with a spontaneous spike used to trigger the stimulus (stimulus artifact at asterisk); the arrow indicates a spontaneous excitatory post-synaptic potential preceding the spontaneous spike. (**B**) Raw record of phrenic nerve (PN) activity and whole cell current clamp recording of an inspiratory-modulated pre-sympathetic neuron from one *in situ* preparation of rat. Perfusion of Alst produced a rapid silencing of this inspiratory-modulated neuron. Note that Alst perfusion revealed the presence of synaptic excitation during inspiration (I) (excitatory post-synaptic potentials - arrows). Abbreviations: PI, post-inspiration; E2, expiratory phase 2. (**C**) Whole cell current clamp recording of the same inspiratory-modulated pre-sympathetic neuron under blockade of fast synaptic transmission. Perfusion of Alst produced again a rapid and reversible silencing of this neuron. (**D**) The same inspiratory-modulated pre-sympathetic neuron labeled *in situ* with biocytin, exhibiting GFP and TH immunofluorescence (a C1 neuron). Scale bar: 20 µm.

**Figure 3:**
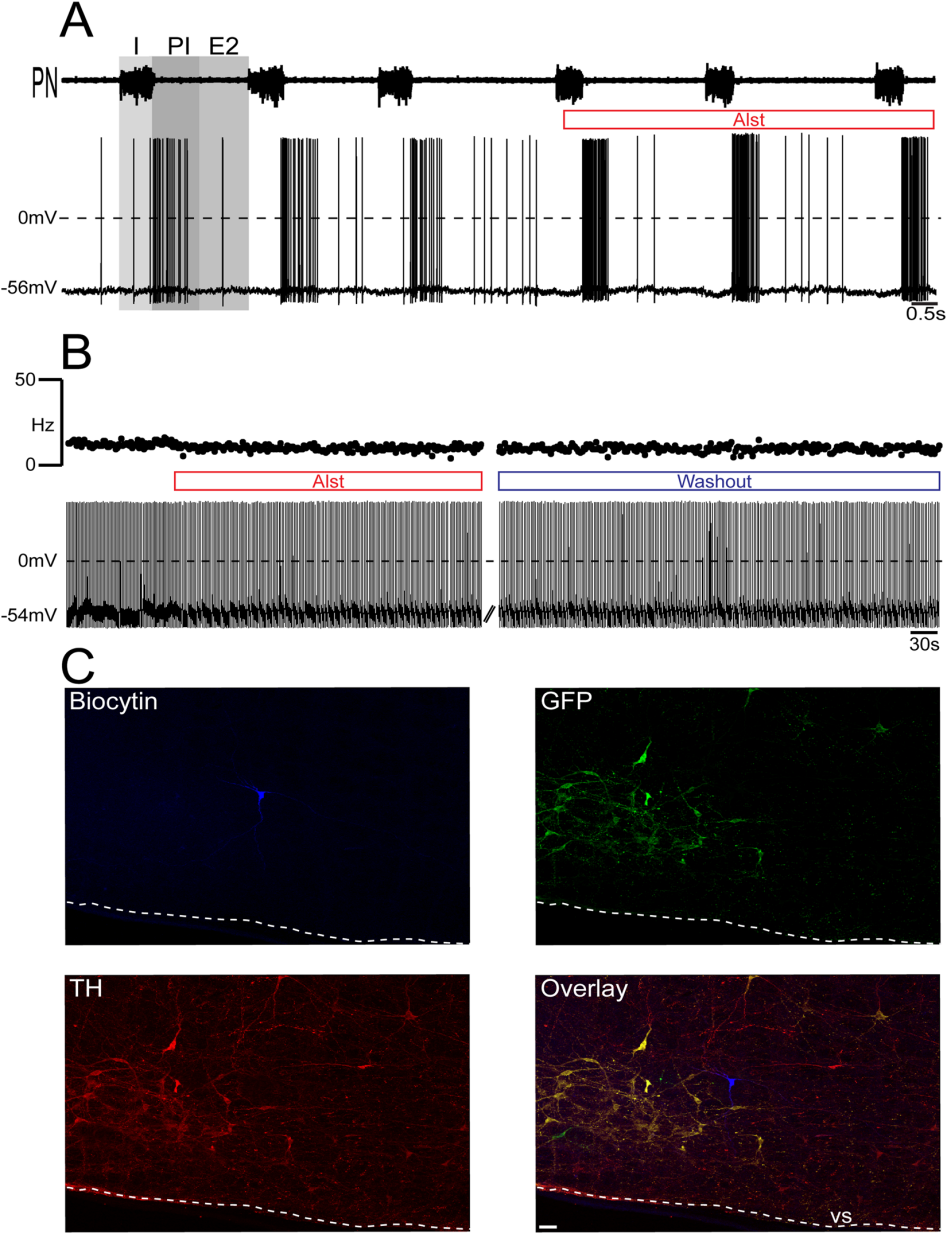
Effect of AlstR activation on the firing frequency of post-inspiratory-modulated RVLM pre-sympathetic neuron recorded *in situ*. (**A**) Raw record of PN activity and whole cell current clamp recording of a post-inspiratory-modulated RVLM pre-sympathetic neuron from one *in situ* preparation of rat. Note that perfusion of Alst did not affect either the firing frequency or the post-inspiratory modulation of this pre-sympathetic neuron. (**B**) Whole cell current clamp recording of the same post-inspiratory-modulated pre-sympathetic neuron under blockade of fast synaptic transmission. Perfusion of Alst did not affect either the intrinsic pacemaker firing frequency or resting membrane potential. (**C**) The same post-inspiratory-modulated pre-sympathetic neuron labeled *in situ* with biocytin, depicting absence of either GFP or TH (a non-C1 neuron) immunofluorescence. Scale bar: 20 um.

Whole cell patch clamp recordings of identified RVLM barosensitive, bulbo-spinal pre-sympathetic neurons (Fig. 2Ai–Aii) were performed in *in situ* preparations of rats, transduced with PRSx8-AlstR-GFP-LVV into C1 region. Without exception, all inspiratory-modulated RVLM bulbo-spinal barosensitive pre-sympathetic neurons recorded were immunopositive for TH (C1 neurons) and expressed GFP (n = 17; Fig. 2D). Arterial perfusion of Alst reversibly eliminated their firing frequency and hyperpolarized their membrane potential either before (from −54.8 ± 0.4 vs −59.3 ± 0.5 mV; p < 0.0001; Fig. 2B) or after blockade of fast synaptic transmission (from −51.9 ± 0.7 to −60.7 ± 0.7 mV; p < 0.0001; Fig. 2C) in all tested neurons. In contrast, all post-inspiratory-modulated RVLM bulbo-spinal barosensitive pre-sympathetic neurons did not express either TH, and hence were not C1 neurons, or GFP after injections of PRSx8-AlstR-GFP-LVV into C1 region (n = 15; Fig. 3C). This was confirmed by demonstrating an absence of effect of Alst on their firing frequency and membrane potential either before [(from 5 ± 1.2 vs 6.3 ± 0.8 Hz) (from – 56.7 ± 1.4 vs −57.3 ± 2.1 mV) Fig. 3A] or after blockade of fast synaptic transmission [(from: 12.1 ± 0.8 to 11.7 ± 1.2 Hz) (from −51 ± 1.2 to −53.5 ± 1.4 mV) Fig. 3B]. These data confirm the effectiveness of targeting the C1 neuronal population using PRSx8-AlstR-GFP-LVV, and that the inspiratory-modulated (but not expiratory-modulated) RVLM pre-sympathetic neurons are the C1 neurons.

### Respiratory-sympathetic responses to suppressing C1 neuronal activity *in situ*

**Figure 4:**
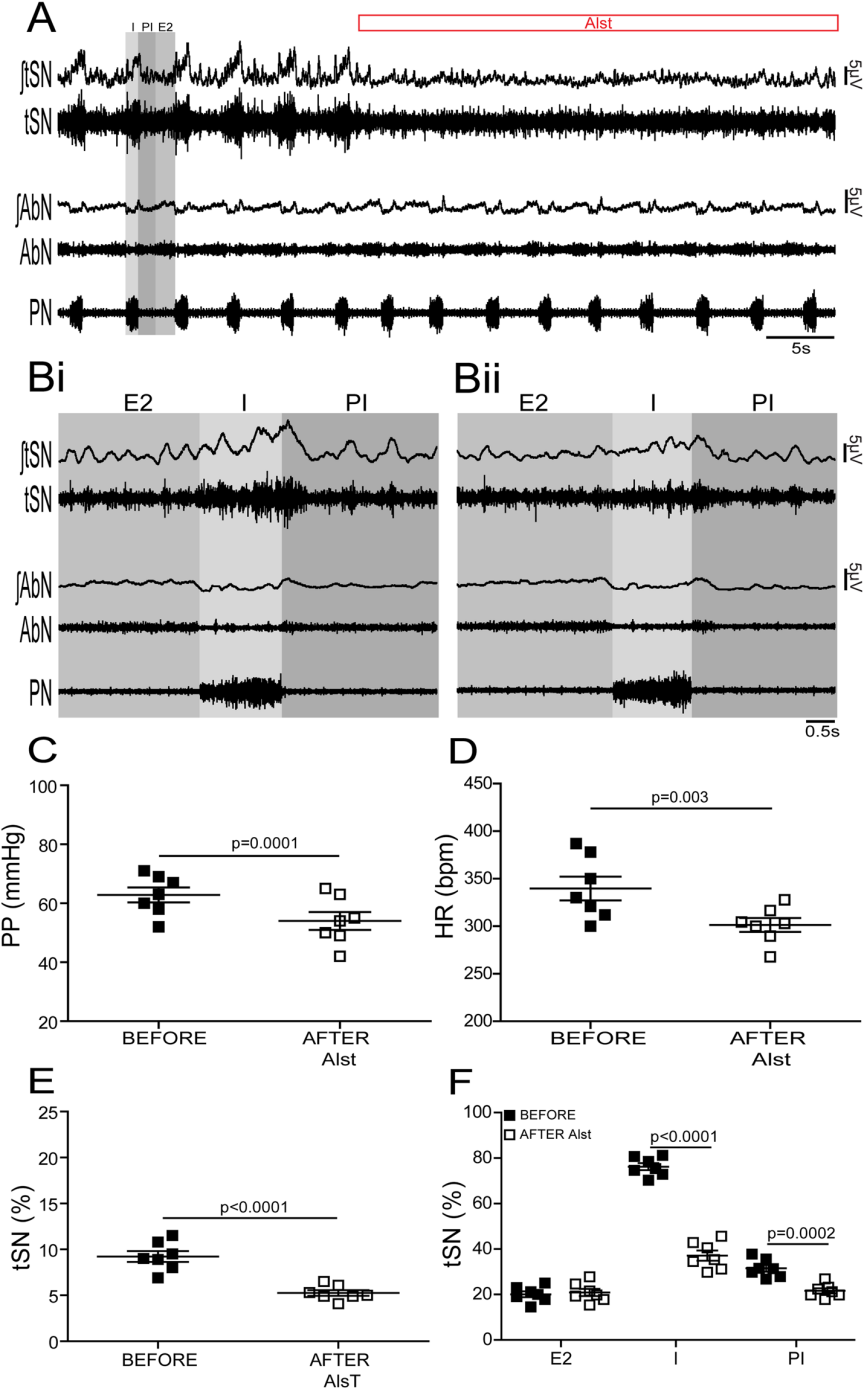
Effects of acute inhibition of C1 neurons on the respiratory and sympathetic activities of *in situ* preparations of control rats. (**A**) Raw and integrated (∫) records of tSN, AbN and PN activities from one *in situ* preparation of control rat transduced with PRSx8-AlstR-GFP-LVV into C1 region before and after arterial perfusion of Alst. PN-triggered averages of tSN and AbN from the same *in situ* preparation before (**Bi**) and after (**Bii**) Alst perfusion. Note that C1 neuronal inhibition reduced the amplitude of the tSN activity during I and PI, but affected neither the inspiratory (PN frequency and amplitude) nor expiratory (AbN) activities. Summary of data showing the changes in the PP (**C**), HR (**D**), baseline tSN activity (**E**) and in tSN activity during I, PI and E2 (**F**) 5 minutes after perfusion of Alst in *in situ* preparations of control rats transduced with PRSx8-AlstR-GFP-LVV into the C1 region.

**Figure 5:**
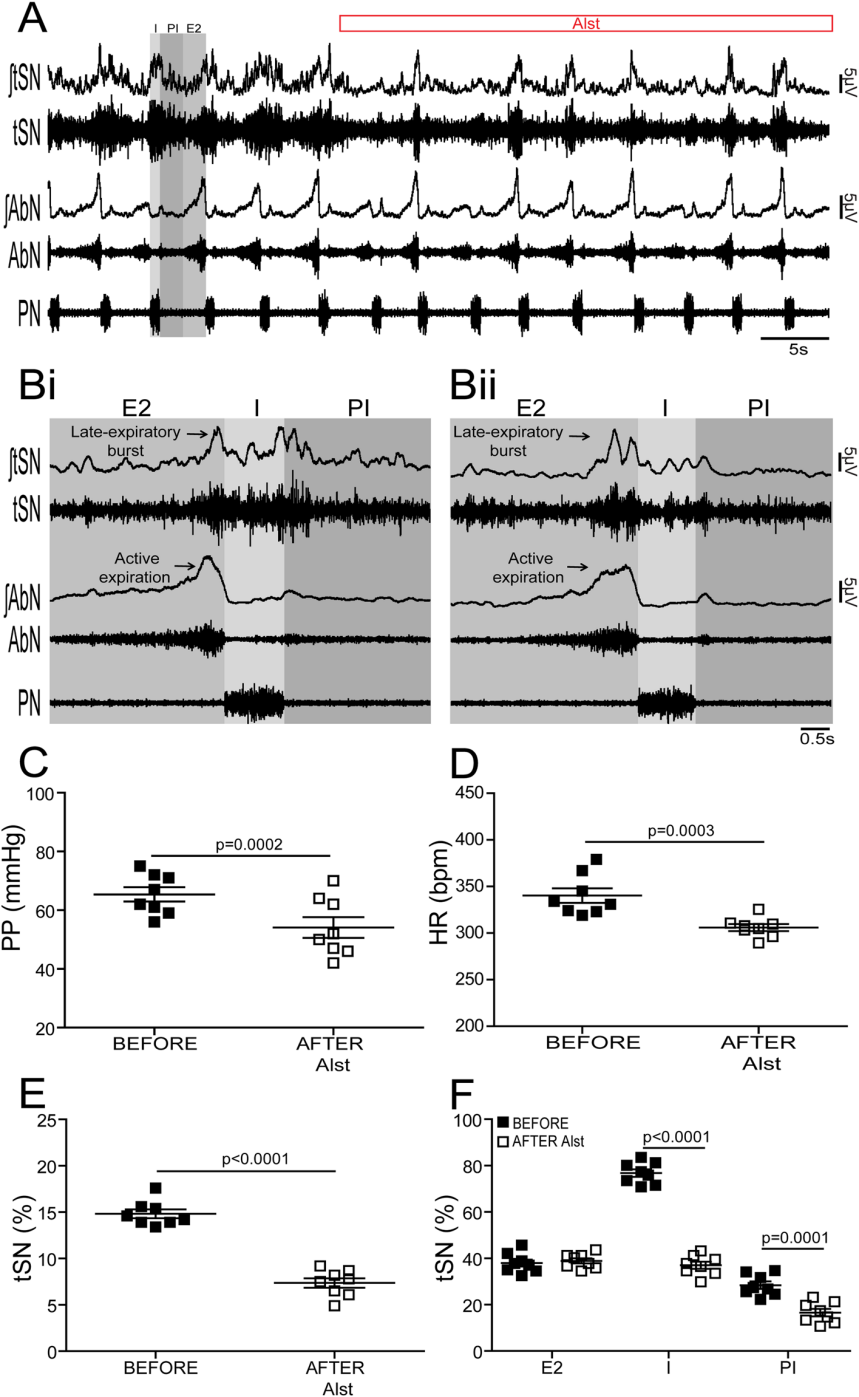
Effects of acute inhibition of C1 neurons on the respiratory and sympathetic activities of *in situ* preparations of CIH rats. (**A**) Raw and integrated (∫) records of tSN, AbN and PN activities from one *in situ* preparation of a CIH rat transduced with PRSx8-AlstR-GFP-LVV into the C1 region before and after arterial perfusion of Alst. PN-triggered averages of tSN and AbN from the same *in situ* preparation before (**Bi**) and after (**Bii**) Alst perfusion. Note that C1 neuronal inhibition reduced the amplitude of the tSN activity during I and PI, but did not affect the inspiratory (PN frequency and amplitude) and the late-expiratory burst of tSN or the AbN active expiration evoked by CIH. Summary of data showing the changes in the PP (**C**), HR (**D**), baseline tSN activity (**E**) and in tSN activity during I, PI and E2 (F) 5 minutes after perfusion of Alst in *in situ* preparations of CIH rats transduced with PRSx8-AlstR-GFP-LVV into the C1 region.

*In situ* preparations of control (n = 7) and CIH (n = 8) rats, in which the RVLM was transduced with PRSx8-AlstR-GFP-LVV, exhibited significant and similar falls in perfusion pressure [PP; (control, from: 62.8 ± 2.5 to 54 ± 3 mmHg; p = 0.0001) (CIH, from: 65.3 ± 2.4 to 54.1 ± 3.5 mmHg; p = 0.0002)], Traube-Hering waves [(control, from 3 ± 0.1 to 0.7 ± 0.05 mmHg; p < 0.0001) (CIH, from 6.4 ± 0.2 to 3.8 ± 0.2 mmHg; p < 0.0001)], heart rate [HR; (control from: 339.7 ± 12.5 to 301.3 ± 7.2 bpm; p = 0.003) (CIH, from: 340.3 ± 7.7 to 306 ± 3.7 bpm; p = 0.0003)] and baseline thoracic sympathetic nerve (tSN) activity [(control, from: 9.2 ± 0.5 to 5.2 ± 0.3%; p < 0.0001) (CIH, from: 14.8 ± 0.4 to 7.3 ± 0.5%; p < 0.0001)] (Figs 4A–E and 5A–E) following arterial perfusion of Alst. Although the frequency [(control, from: 0.29 ± 0.01 to 0.28 ± 0.01 Hz) (CIH, from: 0.28 ± 0.02 to 0.3 ± 0.02 Hz)] and amplitude [(control, from 50.1 ± 1.5 to 52.3 ± 2.1 µV) (CIH, from 51.7 ± 2.2 to 48.9 ± 3 µV)] of phrenic nerve (PN) discharge remained unchanged in the presence of Alst (Figs 4A and 5A), the inspiratory [(control, from 76.2 ± 1.5 to 37.1 ± 2.2%; p < 0.0001) (CIH, from 76.7 ± 1.6 to 37 ± 1.5%; p < 0.0001)] and post-inspiratory [(control, from 31.5 ± 1.5 to 21.5 ± 1.1%; p = 0.0002) (CIH, from 28.3 ± 1.3 to 16.5 ± 1.6%; p = 0.0001)] related tSN activity were decreased significantly (Fig. 4A,B and F; Fig. 5A,B and F). However, in contrast, the late-expiratory related tSN activity, a hallmark phenotype of CIH rats, and the associated active expiration, indexed from recordings from the abdominal nerve (AbN)^10^, were not affected by Alst in CIH rats (late-expiratory bursts in tSN; from 37.9 ± 1.5 to 38.9 ± 1.1%; Fig. 5A,B and F). Alst produced no effect on PP (from 61.7 ± 2.7 to 60.9 ± 2.8 mmHg), HR (from 340 ± 12.7 to 344.6 ± 18.2 bpm) and in baseline tSN (from 10.7 ± 0.5 to 10.5 ± 0.5%) in animals transduced with PRSx8-GFP-LVV (control virus; n = 5) into the C1 region, showing that the Alst-evoked cardiovascular and autonomic responses are mediated by activation of the expressed receptor in C1 neurons.

**Figure 6:**
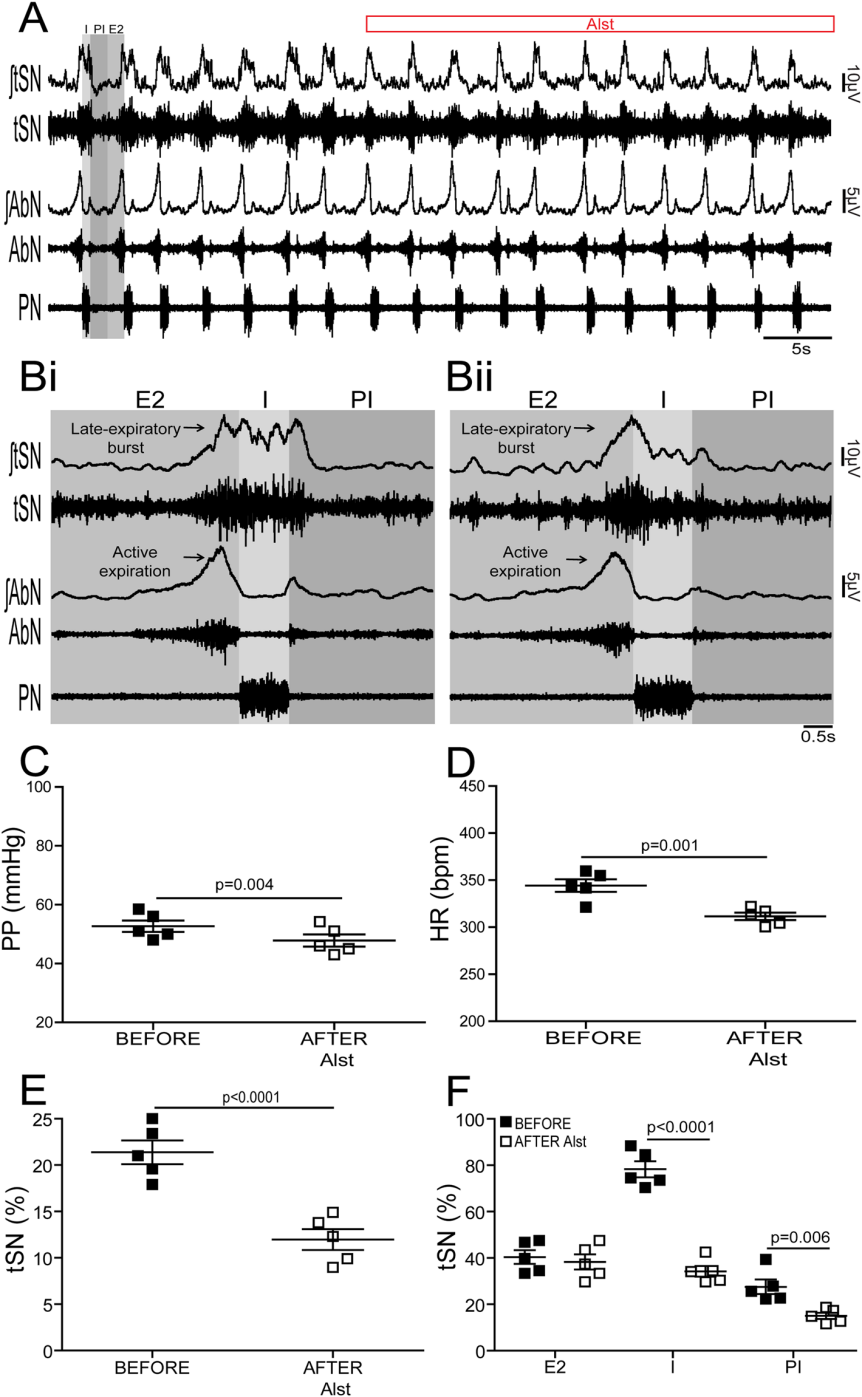
Effects of acute inhibition of C1 neurons on the respiratory and sympathetic activities of *in situ* preparations of control rats under hypercapnia. (**A**) Raw and integrated (∫) records of tSN, AbN and PN activities from one *in situ* preparation of a control rat transduced with PRSx8-AlstR-GFP-LVV into the C1 region under hypercapnia (10% CO_2_) before and after arterial perfusion of Alst. PN-triggered averages of tSN and AbN from the same *in situ* preparation before (**Bi**) and after (**Bii**) Alst perfusion. Note that C1 neuronal inhibition reduced the amplitude of the tSN activity during I and PI, but did not affect the inspiratory (PN frequency and amplitude) and the late-expiratory burst of tSN or the AbN active expiration evoked by hypercapnia. Summary of data showing the changes in the PP (**C**), HR (**D**), baseline tSN activity (**E**) and in tSN activity during I, PI and E2 (**F**) 5 minutes after perfusion of Alst in *in situ* preparations of control rats transduced with PRSx8-AlstR-GFP-LVV into the C1 region under hypercapnia.

**Figure 7:**
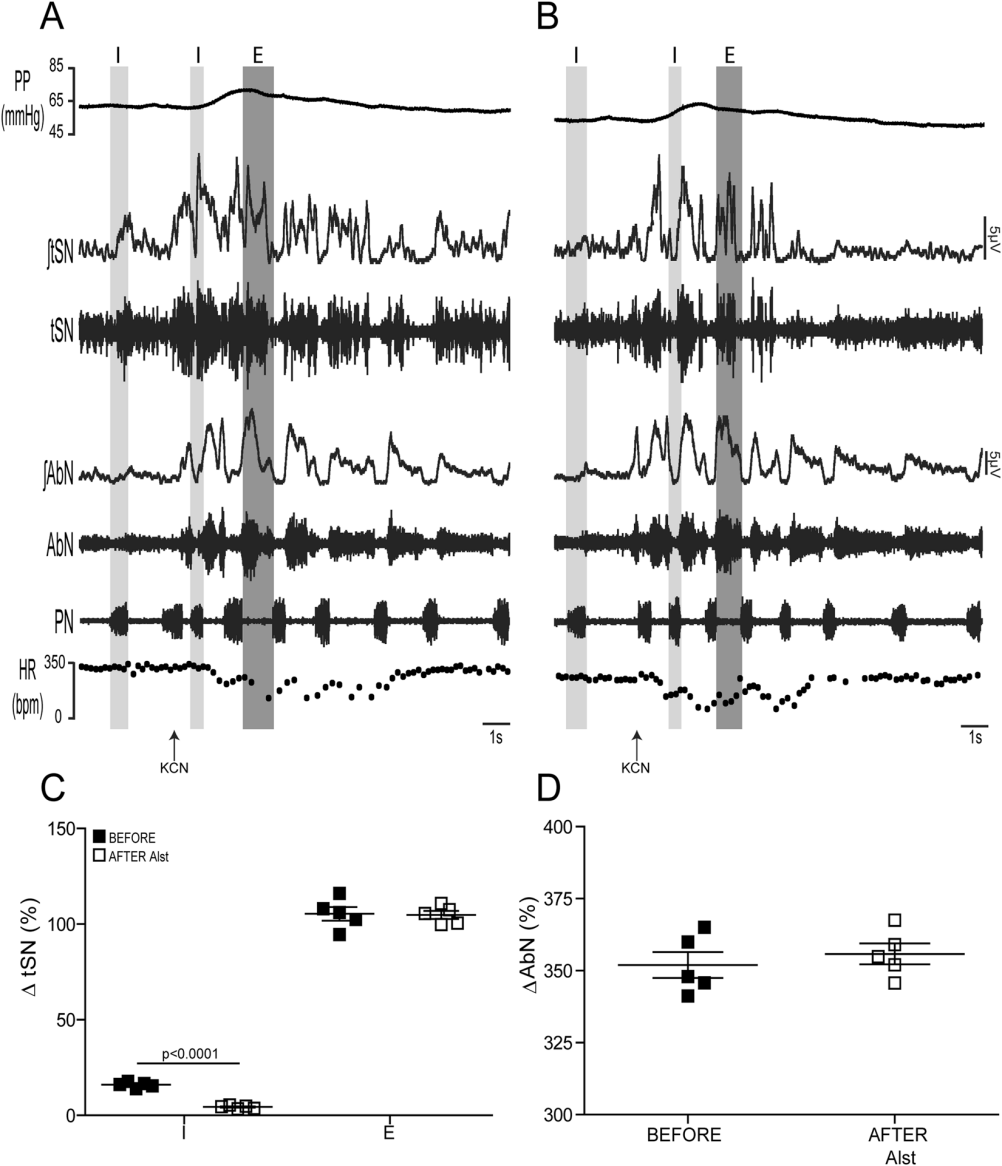
Effects of acute inhibition of C1 neurons on the autonomic, cardiovascular and respiratory responses to peripheral chemoreflex activation of *in situ* preparations of control rats. Raw and integrated (∫) recordings of PN, AbN, tSN activities, and PP and HR of one *in situ* preparation of a control rat, transduced with PRSx8-AlstR-GFP-LVV into the C1 region, illustrating the respiratory, sympathetic, pressure and bradycardic responses elicited by the activation of peripheral chemoreceptors with intra-arterial injection of KCN (0.05%, 50 μl) before (**A**) and after (**B**) arterial perfusion of Alst. (**C**) The percentage of average magnitude of the tSN reflex responses during I, expiration (**E**) and AbN (**D**) responses to peripheral chemoreflex activation in *in situ* preparations of control rats transduced with PRSx8-AlstR-GFP-LVV into the C1 region.

To further validate this conclusion, we suppressed the C1 neurons in *in situ* preparations of control rats transduced with PRSx8-AlstR-GFP-LVV during exposure to hypercapnia (10% CO_2_) or during activation of peripheral chemoreceptors (n = 5), which both enhance the expiratory modulation of sympathetic activity. Despite reductions in PP (from 52.7 ± 1.9 to 47.8 ± 2 mmHg; p = 0.004), Traube-Hering waves (from 5.8 ± 0.3 to 3.4 ± 0.1 mmHg; p = 0.003), HR (from 344 ± 6.6 to 312 ± 3.9 bpm; p = 0.001), baseline tSN activity (from 21.4 ± 1.3 to 12.0 ± 1.1%; p < 0.0001) and in inspiratory (from 78.2 ± 3.4 to 34.2 ± 2.2%; p < 0.0001) and post-inspiratory (from 27.5 ± 3.1 to 15 ± 1.3%; p = 0.006) related tSN activity, Alst neither affected generation of AbN active expiration nor the associated late-expiratory tSN activity (late-expiratory bursts; from 40.4 ± 2.9 to 38.3 ± 3.2%; Fig. 6A–F) evoked by activation of central chemoreceptors. Peripheral chemoreflex activation with potassium cyanide (KCN) produced increases in PN burst frequency, PP, as well as in AbN and tSN activities, and reduced HR (Fig. 7A). In relation to the pattern of the sympathetic response, we verified that the peripheral chemoreflex-evoked tSN bursts occurred preferentially during the expiratory period (ΔtSN during inspiration: 21.3 ± 1.2 vs ΔtSN during expiration: 105.4 ± 3.5%; p < 0.0001; Fig. 7A and C). Arterial perfusion of Alst significantly reduced the tSN inspiratory (ΔtSN from 16 ± 0.6 to 4.3 ± 0.3%; p = 0.0001), but not the tSN expiratory (ΔtSN from 105.4 ± 3.5 to 104.8 ± 2%) responses to peripheral chemoreflex activation (Fig. 7A–C). On the other hand, the AbN expiratory (ΔAbN from 352 ± 4.4 to 355 ± 3.6%), PN inspiratory (ΔPN from 0.38 ± 0.05 to 0.37 ± 0.08 Hz), PP (ΔPP from 18.7 ± 1.4 to 20 ± 2.2 mmHg) and HR (ΔHR from −199 ± 6.4 to −202 ± 8.2 bpm) responses were not affected by Alst (Fig. 7A,B and D). These data suggest that the functional integrity of ventral medullary C1 neurons is not important for the expiratory-related sympathetic bursts generated by CIH or its activation following stimulation of either peripheral or central chemoreceptors.

### Cardiovascular responses to suppressing C1 neuronal activity in conscious rats

**Figure 8:**
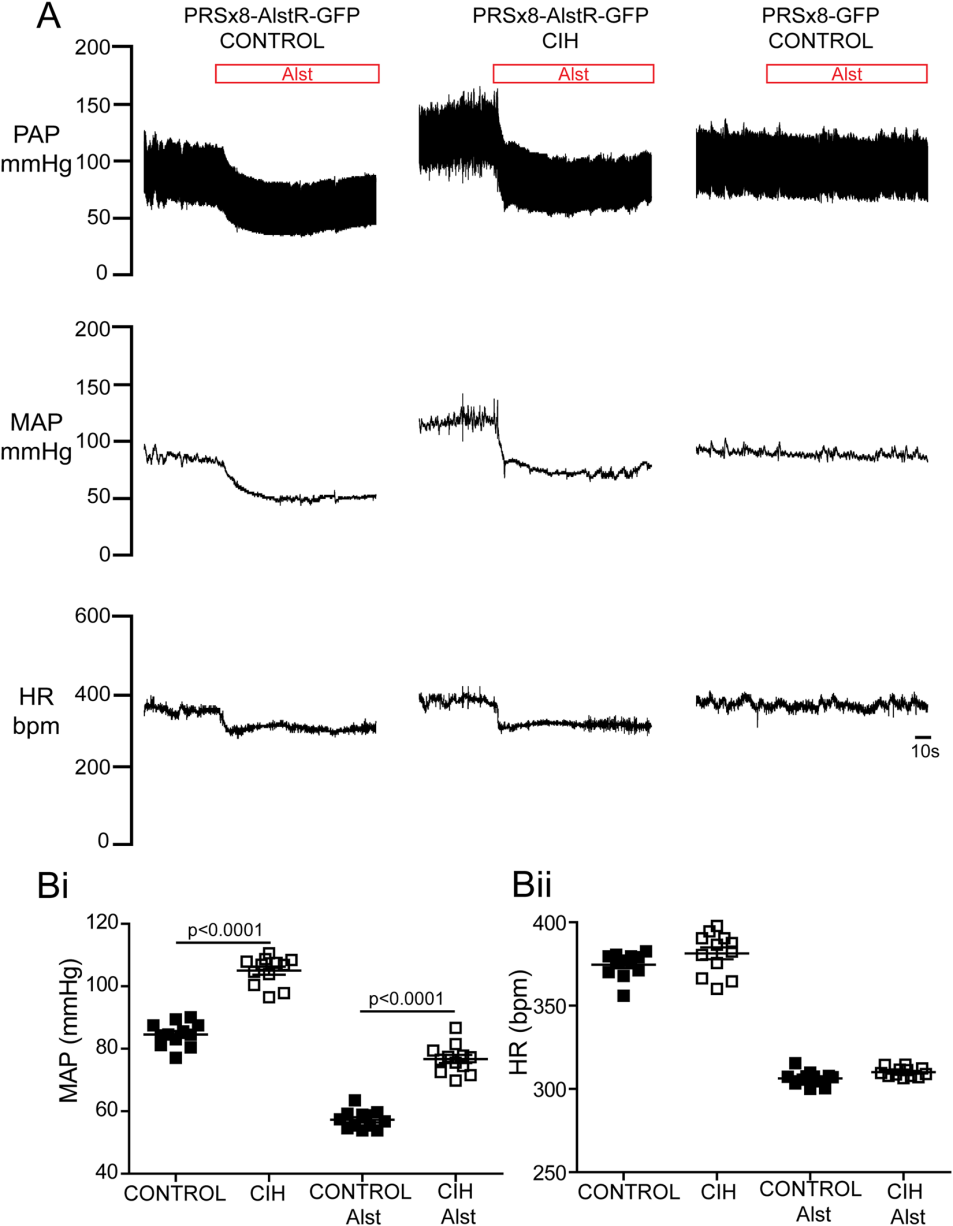
Effects of acute inhibition of C1 neurons on MAP and HR in conscious control and CIH rats. (**A**) Representative recordings illustrating changes in baseline pulsatile arterial pressure [PAP], MAP and HR in response to Alst application into lateral ventricle of control and CIH rats transduced with PRSx8-AlstR-GFP-LVV and one control rat transduced with PRSx8-GFP-LVV into C1 region. Grouped data showing changes in MAP (**Bi**) and HR (**Bii**) 5 min after Alst application in control and CIH rats transduced with PRSx8-AlstR-GFP-LVV into C1 region.

In conscious control rats (n = 12) transduced with PRSx8-AlstR-GFP-LVV in the C1 region, application of Alst (via the lateral ventricle) resulted in a prompt and profound reduction in mean arterial pressure (MAP; from 84.6 ± 1 to 57.2 ± 0.8 mmHg; p < 0.0001) and HR (from 374.6 ± 2.1 to 306.3 ± 1.1 bpm; p < 0.0001; Fig. 8A–Bii). Alst produced no effect on MAP (87.4 ± 1.2 vs 84.6 ± 0.6 mmHg) or HR (378.4 ± 10.5 vs 362.7 ± 13.4 bpm) in animals transduced with PRSx8-GFP-LVV (n = 7; Fig. 8A) into the C1 region. CIH rats (n = 12) presented a higher level of MAP (CIH: 105 ± 1.3 vs control: 84.6 ± 1 mmHg; p < 0.0001), but a similar HR (CIH: 381.4 ± 3.5 vs control: 374.6 ± 2.1 bpm; Fig. 8A–Bii) relative to control rats. Alst application into the lateral ventricle of CIH rats produced reductions in MAP (from 105 ± 1.3 to 76.7 ± 1.3 mmHg; p < 0.0001) and HR (from 381.4 ± 3.5 to 310 ± 0.7 bpm; p < 0.0001), which were of comparable magnitude to those observed in control rats. However, the absolute level of MAP after Alst remained higher in CIH rats compared to controls (CIH: 76.7 ± 1.3 vs control: 57.2 ± 0.8 mmHg; p < 0.0001; Fig. 8A–Bii). These data suggest that the hypertension produced after 10 days of chronic activation of peripheral chemoreceptors (e.g. CIH) was not dependent on the functional integrity of C1 ventral medullary neurons.

## Discussion

In the present study, we used lentiviral vectors to express the inhibitory Drosophila AlstR in a population of sympatho-excitatory pre-sympathetic neurons containing TH (C1 neurons) in the RVLM in order to determine their contribution for the generation of expiratory-related sympathetic activity in CIH rats and also that induced by activation of peripheral or central chemoreceptors in rats. In *in vivo* and *in situ* preparations, acute inhibition of C1 neurons resulted in substantial reductions in arterial pressure, Traube-Hering waves, HR, in baseline and reflex-evoked inspiratory-related sympathetic activity recorded from C1 neurons and from postganglionic nerves. These findings suggest that C1 neurons contribute to the inspiratory-sympathetic coupling and for the maintenance of baseline sympathetic outflow and arterial pressure. However, our data also indicate that pre-sympathetic RVLM C1 neurons are not essential for generating the expiratory-related increases in sympathetic activity evoked by CIH, and activation of either peripheral or central chemoreceptors.

Our histological analysis revealed that ~70% of TH-immunoreactive/Phox2b-negative C1 RVLM neurons located 0–500 µm caudal to the facial nucleus were transduced bilaterally, suggesting that lentiviral targeting results in substantial AlstR-GFP expression among catecholaminergic neurons, which is consistent with that described by others^11,25,31,32^. The expression of AlstR within the RVLM region was also confirmed by the immediate silencing of the firing frequency of whole cell recorded transduced C1 GFP-positive medullary RVLM neurons following Alst applications whereas firing was unperturbed in non-C1 RVLM, GFP-negative cells (Figs 2 and 3). Although the PRSx8 promoter is selective for C1 neurons it is also active in neighboring central chemoreceptors CO_2_/[H^+^]-sensitive Phox2b-positive RTN neurons^30,33^. Indeed, we found a scattering of GFP-expressing neurons showing Phox2b staining in the RTN, caudal to the caudal pole of the facial nucleus. However, all axonal GPF-positive varicosities in the thoracic IML (T8-T10) were TH-immunoreactive (data not shown), suggesting that the transduced Phox2b neurons were not spinally projecting and unlikely to influence sympathetic activity directly. Also, AlstR activation did not change either baseline inspiratory (PN) frequency or amplitude or active expiration recorded in the AbN nor did it affect reflex evoked active expiration following stimulation of either peripheral or central chemoreceptors; these data suggest that RTN Phox2b-positive neuronal population was not infected sufficiently to cause a functional response following Alst application. In addition, RTN Phox2b-positive neurons do not alter sympathetic outflow or arterial pressure when optogenetically stimulated^34^. Therefore, based upon the previous information, we are confident that the observed changes in sympathetic outflow, HR and arterial pressure in response to AlstR activation in both *in vivo* and *in situ* are mediated by inhibition of RVLM C1 neurons.

The role of sympatho-excitatory RVLM C1 neurons in regulating baseline respiratory-related sympathetic activity, Traube-Hering waves and cardiovascular function was addressed previously in normotensive and spontaneously hypertensive (SH) rats^11,24,25^. The experimental approach used in the present study caused a significant reduction in baseline sympathetic outflow, by suppressing mainly inspiratory-modulated activity, but also its small peak during post-inspiration, HR, Traube-Hering waves and arterial pressure in conscious and *in situ* preparations. Recent studies involving selective optical stimulation of genetically targeted RVLM C1 neurons confirmed that their activation increased respiratory-related sympathetic activity^31^ and MAP^32^. However, our observation of cardiovascular effects differs from recent work reporting very little change in MAP after acute C1 neuronal optical inhibition in conscious rats^35^. Both approaches have certain limitations and this discrepancy may reflect differences in strains (Wistar vs Sprague Dawley) and age (juvenile vs adult) of rats, topographical distribution of transfected C1 neurons (faster vs slowly conducting transfected C1 neurons^29^), methods for neuronal manipulations (long-lasting pharmacogenetic inhibition vs acute optogenetic inhibition), conscious state, and the amount of stress or recent manipulations/anesthesia (e.g. the use of anesthesia was used to connect the optical fibers one hour before experiments^35^) of rats.

RVLM neurons exhibit different patterns of activity entrained by the respiratory pattern generator, with peaks of activity modulated by either I or PI^19,22,36–38^. We showed that Alst inhibited TH-positive inspiratory-modulated, but not TH-negative post-inspiratory-modulated RVLM bulbo-spinal barosensitive pre-sympathetic neurons. We propose that the peak of tSN activity during post-inspiration is also generated by RVLM inspiratory-modulated pre-sympathetic C1 neurons. The conduction velocity delays both in the pathway from the RVLM site to IML and delays in the efferent nerve traffic to the site of tSN recording (T8-T10) may shift the peak of tSN activity from inspiration to post-inspiration. These properties may explain why the inhibition of inspiratory-modulated RVLM C1 neurons using Alst also reduced the peak of tSN activity during post-inspiration. The expiratory-related sympathetic discharge (tSN late-expiratory burst) evoked by CIH, activation of peripheral or central chemoreceptors, was associated with a selective enhancement of the frequency of spontaneous excitatory post-synaptic events and the firing frequency of post-inspiratory-modulated non-C1 RVLM neurons, in the same phase of the respiratory cycle (late-expiratory activity)^19^. Given that MAP remained higher after C1 neuronal inhibition in CIH rats versus controls, this suggests that an important component of the CIH-evoked expiratory-related sympathetic activity and hypertension is caused by the bulbo-spinal non-catecholaminergic post-inspiratory-modulated RVLM neuronal activity; a finding not dis-similar to that we described in the SH rats^36^. In this regard, Menuet *et al*.^11^ demonstrated that deleting a large proportion of C1 neurons attenuated, but did not prevent, the development of hypertension in SH rats, suggesting that non-C1 neurons may also contribute to the generation of sympathetic over activity and high blood pressure in this animal model. We acknowledge that we cannot rule out other brainstem sources of expiratory-related neuronal firing from, for example, the A5 cell group of the pons^39^, spinal pre- or even post-ganglionic neurons^40^.

Activation of central or peripheral chemoreceptors evoked significant increases in tSN during inspiration, but mainly during expiration^7,8,41^. Although, there is evidence that hypercapnia increases discharges of C1 neurons *in vivo* ^42^, recent data suggest that RVLM C1 neurons do not appear to be responsible for the increases in sympathetic outflow evoked by central actions of CO_2_^25^. In this regard, following C1 neuronal inhibition in the present study, the baseline level of tSN activity remained higher during hypercapnia than during normocapnia, even after reductions in inspiratory- and post-inspiratory-related sympathetic activity. Expiratory increases in sympathetic activity in response to activation of central chemoreceptors were not affected after C1 neuronal inhibition. The parafacial Respiratory Group (pFRG) has been proposed to be one of the sources of this expiratory modulation^8^. The pFRG contains neurons that are silent during breathing at normoxia and normocapnia^43^, but evoked during activation of central^44,45^ or peripheral chemoreceptors^46^, exhibiting a pattern of activity that is strongly correlated with AbN active expiration and the late-expiratory related sympathetic discharges. These late-expiratory neurons of the pFRG could provide excitatory drive not only to expiratory abdominal motoneurons in the spinal cord to produce active expiration^45^, but also to bulbo-spinal barosensitive post-inspiratory-modulated non-C1 pre-sympathetic RVLM neurons, culminating in increases of sympathetic activity correlated with AbN active expiration observed during hypercapnia and hypoxia. In this regard, Dempsey *et al*.^47^ identified monosynaptically connected input neurons from pFRG to RVLM bulbo-spinal pre-sympathetic neurons, which may contribute to the generation of sympatho-excitation during late-expiration under conditions of enhanced central respiratory drive. Future electrophysiological experiments are needed to evaluate the contribution of pFRG late-expiratory neurons for the generation of expiratory-related sympathetic activity observed in rats under hypercapnia, hypoxia and after CIH to ascertain whether this neural coupling mechanism is common to other forms of neurogenic hypertension.

As far as the C1 neurons appear to be critically involved in the generation of the baseline respiratory modulation of sympathetic activity^11,25^, we tested the possibility that this neuronal population is involved in the processing of the inspiratory- and expiratory-modulated sympathetic reflex responses. We verified that inhibition of C1 neurons reduced the inspiratory, but not the expiratory, sympatho-excitatory responses to peripheral chemoreflex activation. Therefore, as observed in chronic intermittent activation of peripheral chemoreceptors (e.g. CIH), these findings indicate that the C1 neurons play no major role in modulation of the expiratory sympathetic reflex response to acute activation of the carotid bodies using KCN. Previous studies from our laboratory demonstrated that depression of post-inspiratory activity (elicited by inhibition of either the nucleus tractus solitarii or Bötzinger Complex) reduced the magnitude of the expiratory-related sympatho-excitatory response to peripheral chemoreflex activation^7,48^. We propose that inhibitory RVLM-projecting neurons in the caudal ventrolateral medulla, with expiratory-modulated activity under resting conditions and inhibited during activation of peripheral chemoreceptors^49^, may also disinhibit RVLM neurons at expiration during hypoxia. These proposed connections are not yet proven and still require further experimental verification.

In summary, our data confirm that the C1 neuronal population significantly contributes to the maintenance of both sympathetic tone, through its inspiratory modulation, and arterial pressure. Although C1 neurons do not appear to mediate the expiratory-related sympathetic activity and hypertension evoked by CIH, this study provides important guidance for further studies seeking to understand brainstem mechanisms underlying increases in sympathetic outflow and cardiovascular dysfunctions which accompany disruptions in gas exchange in patients with obstructive sleep apnea(OSA) and hypertension, for example. Our study supports an important role of expiratory-related oscillations in the generation of active expiration and their significant contribution for the development of hypertension and sympatho-excitatory responses to cardio-respiratory reflex activation under conditions of increased central respiratory drive. The neurons responsible for these effects may provide a novel and highly selective target for treatment of hypertension in conditions of CIH and perhaps in patients with OSA.

## Methods

### Animals

The experiments were performed on male Wistar rats provided by the Animal Care Facility at the Ribeirão Preto campus of the University of São Paulo (USP), Brazil. All experimental protocols were approved by the Institutional Ethics Committee for Animal Experimentation at the School of Medicine of Ribeirão Preto, USP (protocols 093/2009 and 064/2010). All methods were carried out in accordance with The Principles of Laboratory Animal Care (NIH publication no. 85Y23, revised 1996). Animals were housed with a 12 h light/dark cycle at a constant temperature (22 ± 1 °C) with *ad libitum* access to standard rat chow and water.

### Viral vectors

The LVV system used here was HIV-1-derived and pseudo-typed with the VSV-G envelope^50^. The plasmids pTYF-PRSx8-AlstR-IRES2-GFP and pTYF-PRSx8-IRES2-GFP were cloned into the LVV. Titres of PRSx8-AlstR-GFP-LVV and the control virus (PRSx8-GFP-LVV) were between 1 × 10^9^ and 1 × 10^10^ pfu. Viral concentration and titration were performed as described in detail previously^50^.

### *In vivo* gene transfer

Post-weaned male rats (50–55 g) were anaesthetized with a ketamine (75 mg kg^−1^ i.p.)/xylazine (5 mg kg^−1^ i.p.) mixture. The depth of anesthesia was checked at regular intervals (20–30 min) by assessing the withdrawal reflex response to noxious pinching of the tail or hind paw. Animals were placed in a stereotaxic frame (tooth bar −3.5 mm below the inter-aural line; David Kopf, Tujunga, USA) and two microinjections (different rostrocaudal levels separated by 300 μm) per side of either PRSx8-AlstR-GFP-LVV or PRSx8-GFP-LVV (50 nl each, over 5 min) were delivered into RVLM (Picospritzer II; Parker Instruments, Cleveland, USA). The injection pipette was angled at 25° and injections were made −3.7 mm ventral from calamus scriptorius and ± 1.7 mm lateral from the midline. Post-surgery, rats were treated with one prophylactic dose of analgesic and antipyretic flunixin meglumine (1 mg kg^−1^; Schering-Plough, Rio de Janeiro, Brazil) and 0.1 ml of veterinary antibiotic (1.2 million i.u.; Fort Dodge, Campinas, Brazil) via intramuscular injections.

### Chronic Intermittent Hypoxia

Five days after the LVV injections, rats were divided into 2 groups: CIH treatment (6% of O_2_ for 30–40 s, every 9 minutes and 8 hours a day) and normoxia (20.8% of O_2_) for 10 days as this CIH protocol generates forced expiration and increases in expiratory-related burst of sympathetic activity^19^.

### Cardiovascular measurements in conscious animals

At the end of the hypoxic or normoxic protocols, animals were re-anesthetized. A stainless-steel guide cannula (13 mm long, 0.6 mm o.d., 0.4 mm i.d.) was implanted into the lateral cerebral ventricle (−0.6 mm to Bregma, 1.5 mm lateral to the midline and −3.6 mm ventral to dura mater) and a catheter (PE-10 connected to PE-50; Clay Adams, Parsippany, USA) inserted into the abdominal aorta, through the femoral artery, for pulsatile arterial pressure measurements. MAP and HR were processed from PAP by a data acquisition system. Post-surgery, rats were treated again with analgesic and antipyretic and veterinary antibiotic. Two days later, the arterial catheter was connected to a pressure transducer (MLT0380; ADInstruments, Sidney, Australia), and in turn, to an amplifier (ML221; ADInstruments). PAP was recorded in conscious, freely moving rats under normoxic conditions (1 kHz; Chart Pro, PowerLab 4/25, ML845; ADInstruments). Alst [2 mM, 10 µl; Phoenix Pharmaceuticals, Inc., Burlingame, USA^33^] was administered intracerebroventricullarly [(25 µl syringe; Hamilton Company, Reno, USA) (needle 33-gauge; Small Parts, Miami Lakes, USA)]. The correct placement of the guide cannula was confirmed at the end of the experiment by injection of Evans Blue (2% in 10 μl; Sigma-Aldrich, St. Louis, USA) and its visible presence in the intracerebroventricular system.

### *In situ* perfused preparations of rats and neurophysiological recordings

Control and CIH rats were prepared for *in situ* perfused preparation^51^ at the end of the CIH and control protocols. The ventral medullary surface was exposed using a ventral approach^19^ for neuronal recordings in the RVLM. Peripheral chemoreceptors were stimulated by injections of KCN (0.05%, 50 μl; Sigma-Aldrich) into the descending aorta via a side branch of the perfusion cannula. Central chemoreceptors were stimulated by hypercapnia raising CO_2_ to 10% with 90% O_2_ in control rats after acute bilateral denervation of carotid bodies. Alst (1 µM) was added to the perfusate and fresh perfusate was used to washout the peptide^33^.

PN, tSN and AbN nerves and the ECG were recorded simultaneously using bipolar electrodes mounted on separate 3D micromanipulators (YOU-1; Narishige, Tokyo, Japan). Left PN discharges were recorded from the central end. Right thoracic/lumbar AbN (T13-L1) were isolated from abdominal muscles and cut distally, and their central activity was recorded. The efferent activity of the tSN was recorded from the sympathetic chain (T8-T10). All signals were amplified, band-pass filtered (0.1–5 kHz; 1700 amplifier, A-M Systems, Sequim, USA) and acquired with an A/D converter (5 kHz; CED 1401, Cambridge Electronic Design, Cambridge, UK) controlled by a computer running Spike 2 software (Cambridge Electronic Design). All nerves were recorded in absolute units (μV) and analyses were performed off-line from rectified and integrated signals (time constant: 50 ms). Baseline PN burst frequency was assessed. Active expiration was determined when the AbN activity at the end of expiration was bigger (µV) than the AbN activity earlier in expiration. Based upon absolute values of tSN (µV), we determined a percentage scale, in the range from 0 to 100, in order to compare the levels of baseline tSN across animals during different experimental conditions in control and CIH rats. This percentage scale was determined for each preparation and was based on the maximal activity observed during ischemia (pump-off) as 100% and the electrical noise level as 0% obtained at the end of each experiment after the death of the preparation. PN-triggered averages of tSN were generated from 1–2 min epochs and divided into three parts: I (coincident with inspiratory PN discharge), PI (first half of expiratory phase) and E2 (second half of expiration). The tSN activities during each respiratory phase were assessed by the measurement of individual area under the curve and normalized by the total area (100%), i.e. sum of areas. PN frequency, HR and PP responses to chemoreflex activation were assessed by the difference between baseline and the peak of response observed after the stimulus (ΔPN, ΔHR and ΔPP). The tSN (during inspiration and expiration) and AbN expiratory responses to peripheral chemoreflex activation were assessed by the measurement of the area under the curve, in a time window of ≤ 10 s after the stimulus, and expressed as percentage values (ΔtSN and ΔAbN in percentage) in relation to the activity before the stimulus.

Blind whole cell patch-clamp recordings were obtained from RVLM pre-sympathetic neurons with electrodes filled with a solution containing in mM: 130 K-gluconate; 4.5 MgCl_2_; 14 trisphosphocreatine, 10 HEPES; 5 EGTA; 4 Na-ATP; 0.3 Na-GTP; 0.2% biocytin (Molecular Probes, Grand Island, USA)^36,52^. RVLM pre-sympathetic neurons were characterized by their inhibitory response to baroreflex activation (by electrically stimulating ADN; 0.2 ms, 50 Hz, 2 s) and the presence of antidromic responses evoked by stimulation (0.2 ms; 1 Hz) of spinal segments T8-T10 (DS2A, Digitimer, Welwyn Garden City, UK)^53^. Current-clamp experiments were performed using an Axopatch-200B integrating amplifier (Molecular Devices, Sunnyvale, USA) and pClamp acquisition software (version 10.0, Molecular Devices). All data were low-pass filtered at 5 KHz and digitized at the rate of 20 KHz (Digidata 1550, Molecular Devices). The intrinsic pacemaker property of RVLM pre-sympathetic neurons was evaluated in the presence of blockers of fast synaptic transmission in the perfusate (2.5–6.0 mM kynurenic acid, 20 µM bicuculline and 1 µM strychnine; Sigma-Aldrich). A liquid junction potential of 15 mV was corrected off-line. All-points histograms were constructed using 1 min of membrane potential values recorded in each experimental condition and the resting membrane potential value was considered to be the value at which the cells spent most of the time as defined previously^19^.

### Histology

Immunohistochemistry was performed on sections from the brainstem and spinal cord of control and CIH rats to evaluate the location of labelled RVLM neurons and detection of: TH, Phox2b, ChAT and GFP expression following viral gene transfer. Animals used for neuronal tracing studies were sacrificed 6 weeks after virus injections to optimize fluorophore labeling of axonal projections to the IML. Standard perfusion and post-fixation protocols were performed as previously published^54^. Immunohistochemistry protocols were performed as previously published for fluorescence immunohistochemistry^54^. The primary antibodies used here were as follows: mouse anti-TH (1:5000; Millipore), chicken anti-GFP (1:5000; Abcam, Cambridge, UK), goat anti-ChAT (1:1000; Millipore) and rabbit anti-Phox2b (1:800; gift from J.-F. Brunet). The secondary antibodies for fluorescence immunohistochemistry were: Cy3-conjugated donkey anti-rabbit (1:250; Jackson ImmunoResearch Laboratories, Inc., West Grove, USA), AlexaFluor-647 goat anti-mouse (1:250; Molecular Probes), AlexaFluor-488 donkey anti-chicken (1:250; Molecular Probes), AlexaFluor-647 donkey anti-goat (1:250; Molecular Probes), and Cy3-conjugated streptavidin (1:1000; Jackson ImmunoResearch Laboratories, Inc.). Images were collected on a Leica TCS SP5 confocal microscope (Buffalo Grove, USA) equipped with 488, 543 and 633 nm laser lines and tunable emission wavelength detection. Neurons were counted in every one in three series of 40 μm coronal sections.

### Statistical Analyses

Results are expressed as means ± SED and two-way ANOVA followed by Bonferroni’s post hoc test were used to compare tSN activity among phases of the respiratory cycle in *in situ* preparations, MAP and HR in conscious rats exposed to CIH and control conditions before and after Alst. Student’s paired t test was used for direct comparisons of neuronal activity, PP, Traube-Hering waves, HR, tSN, PN and AbN before and after Alst in the same experimental group (GraphPad Prism 4, La Jolla, USA).
